# ClaPEPCK4: target gene for breeding innovative watermelon germplasm with low malic acid and high sweetness

**DOI:** 10.1080/21645698.2025.2452702

**Published:** 2025-01-14

**Authors:** Congji Yang, Jiale Shi, Yuanyuan Qin, ShengQi Hua, Jiancheng Bao, Xueyan Liu, Yuqi Peng, Yige Gu, Wei Dong

**Affiliations:** a State Key Laboratory of Cotton Biology, School of Life Sciences, Henan University, Kaifeng, China; b School of Life Science, Henan University, Kaifeng, Henan, People’s Republic of China

**Keywords:** Fruit development, gluconeogenesis, malic acid, *PEPCK*, watermelon

## Abstract

Malic acid markedly affects watermelon flavor. Reducing the malic acid content can significantly increase the sweetness of watermelon. An effective solution strategy is to reduce watermelon malic acid content through molecular breeding technology. In this study, we measured the TSS and pH of six watermelon varieties at four growth nodes. The TSS content was very low at 10 DAP and accumulated rapidly at 18, 26, and 34 DAP. Three phosphoenolpyruvate carboxykinase (*PEPCK*) genes of watermelon were identified and analyzed. The *ClaPEPCK4* expression was inversely proportional to malate content variations in fruits. In transgenic watermelon plants, overexpressing the *ClaPEPCK4* gene, malic acid content markedly decreased. In the knockout transgenic watermelon plants, two SNP mutations and one base deletion occurred in the *ClaPEPCK4* gene, with the malic acid content in the leaves increasing considerably and the PEPCK enzyme activity reduced to half of the wild-type. It is interesting that the *ClaPEPCK4* gene triggered the closure of leaf stomata under dark conditions in the knockout transgenic plants, which indicated its involvement in stomatal movement. In conclusion, this study provides a gene target *ClaPEPCK4* for creating innovative new high-sweetness watermelon varieties.

## Introduction

Watermelon contains various nutritional components such as protein, glutamic acid, vitamins, and carotenoids, which can be consumed to supplement the body-required nutrients.^1^ Watermelon quality is an important factor affecting its the sales volume. As living standards improve, the pursuit of health has become a priority. Developing new watermelon varieties with high sweetness and low sugar content is a key breeding direction. Normally, fruits with high sugar to acid ratios tend to have a sweeter taste, while fruits with low sugar to acid ratios tend to have a more sour taste.^2^ Maintaining the sugar content of watermelon and reducing the organic acid content can effectively solve the problem of improving the sweetness of watermelon taste.

Fruit organic acids are components affecting fruit quality and have been studied in many fruits such as apricots, citrus, apples, and pears.^3–6^ The organic acids in the fruit exhibit a pattern of first accumulation and then consumption during development.^7,8^ However, during the development of apples, there are significant differences in the content of malic acid and citric acid at different stages of development.^9^ Therefore, the synthesis and degradation of organic acids are one of the important factors affecting fruit quality. In watermelon, soluble sugars primarily comprise fructose and sucrose, while organic acids mainly comprise malic and citric acids.^10^ While sugar transporters are positively correlated with sugar content, malic and citric acids are strongly correlated with malate and citric acid transporters.^2^ The organic acids in watermelon flesh also accumulate in the early stages of development and decrease in the later stages of maturity.^11^ Although the negative correlation between organic acid content and watermelon fruit quality is known, few studies have explored changes in the basic metabolism and synthesis of sugars or secondary compounds in watermelon.

Malic acid is converted to oxaloacetic acid by malate dehydrogenase or to phosphoenolpyruvate by phosphoenolpyruvate carboxykinase (PEPCK). PEPCK, as a key enzyme, exists widely in various animals, plants, and microorganisms. In plants, PEPCK catalyzes oxaloacetate decarboxylation to phosphoenolpyruvate. PEPCK is involved in various plant growth and development processes, including promoting seed germination, concentrating CO_2_ in C4 and CAM photosynthesis, and converting organic acids into sugars. Its involvement in gluconeogenesis and the metabolism of ammonia or asparagine in maize has been reported.^12^ In *Arabidopsis*, *PEPCK* is also involved in the closure of leaf stomata.^13^ *PEPCK* overexpression in tomatoes can promote soluble sugar accumulation and decrease malate content, while its disruption has the opposite effect.^14,15^ PEPCK overexpression in strawberries affects the citric acid content by inhibiting citric acid synthase activity.^16^ *PEPCK's* appearance in blackberries, grapes, peaches, and other fruits, which contributes to malate reduction, also indicates its marked contribution to gluconeogenesis and malate metabolism.^17–20^ However, the contribution of *PEPCK* to malic acid metabolism in watermelon has not yet been determined.

In traditional breeding, by detecting the organic acid content of the parents, hybridization and selection can be effectively carried out in order to obtain new varieties with low organic acid content in the offspring. Due to the narrow diversity of watermelons, traditional hybridization methods cannot produce exceptionally sweet watermelons. This study verifies from a molecular perspective whether the expression levels of organic acids and the *PEPCK* genes conform to genetic laws by detecting the parents and their F1 hybrid offspring. We identified the *PEPCK* family members, conducted bioinformation analysis, explored the molecular mechanism of *PEPCK* genes overexpression in watermelon, and created innovative new germplasm resources with low acidity and high sweetness using molecular technology.

## Materials and Methods

### Plant Material

Two groups of parents and their offspring were used as experimental materials provided by the watermelon research group at the College of Life Sciences, Henan University. Watermelon germplasm “V206,” “V208,” “P05,” and “P15” (*Citrullus lanatus* subsp. *vulgaris*) were self-pollinated for 10 generations to obtain stable phenotypes since 2014. The first group is Group V, with watermelon germplasm “V206” serving as the female parent, “V208” as the male parent, and “F1203” as their F1 offspring. The second group is Group P, with “P05” serving as the female parent, “P15” as the male parent, and “F1251” as their F1 offspring.

Use plug trays for seedling cultivation in the incubator and cultivated under 28°C 16-h light, 25°C 8-h dark, and 70% humidity. After 1 month, transplant watermelon seedlings to the greenhouse. After 1 month, we performed hybrid pollination and watermelon fruits 10, 18, 26, and 34 d after pollination (DAP) were used as experimental materials. The experimental materials were planted in Rice Township, Longitude: 114.303090°, Latitude: 34.872040°, Kaifeng City, Henan Province. Part of the central flesh of the watermelon was frozen in liquid nitrogen and stored in a − 80°C refrigerator for follow-up experiments.

### Measuring Malate and Citric Acid Content in Watermelons

The malate and citric acid contents in watermelon pulp were determined using high-performance liquid chromatography (HPLC).^21^ To extract organic acids, 1 g watermelon pulp was ground and mixed with 5 mL of ddH_2_O. The mixture was then heated in a water bath at 80°C for 30 min. After heating, the volume was adjusted to 10 mL, centrifuged at 4000 rpm for 10 min, and filtered through a 0.45 μm filter membrane. The filtered sample was then analyzed using HPLC. The mobile-phase solution was a 10 mm KH_2_PO_4_ solution (pH = 2.65), and the flow rate was 0.8 mL/min, the UV absorbance was 210 nm, and the sample volume was 20 μL.

### RNA Isolation and Quantitative Real-Time PCR

The Polysaccharide/Polyphenol Plant Total RNA Mini Kit (Gene Better, Beijing, China) was used to isolate the total RNA from the samples collected at each fruit developmental stage. The isolated RNA content was quantified using a NanoDrop (Thermo Scientific, Shanghai, China), and RNA quality was assessed using agarose gel electrophoresis. The high-quality total RNA (1 μg) served as a template for synthesizing first-strand cDNA using the SweScript RT II First Strand cDNA Synthesis Kit (With gDNA Remover) (Servicebio, Wuhan, China). The cDNA was analyzed in qRT-PCR assays performed using LightCycler480 II (Roche, Shanghai, China). The reaction mixtures included 10 μL SYBR Premix Ex Taq II (TaKaRa, Beijing, China) and specific primers (Table S1). Analyses were completed with three biological replicates, each comprising three technical replicates. The relative expression levels were calculated using the 2^−∆∆Ct^ method.

### Identification of PEPCK Genes in Watermelon Genome and Peptide Property Predictions

Homologous *PEPCK* sequences were searched via BLASTp on the CuGenDBv2 (http://www.cucurbitgenomics.org/) website^22–24^ The obtained sequences were then searched for the presence of the PEPCK-specific domains [PEPCK_ATP (PF01293)] through PFAM (https://www.ebi.ac.uk/interpro/entry/pfam/#table). The Simple HMM Search function of TBtools^25^ was used to screen *PEPCK* sequences in the watermelon genome. Subsequently, the retrieved candidates were screened through the Conserved Domain Database (CDD)^26^ and Simple Modular Architecture Research Tool (SMART) for validation. Corresponding genomic DNA sequences, coding DNA sequences (CDS), and other related sequences were retrieved from the watermelon genome v2.^24^ Various peptide properties, such as molecular weight, amino acid length, isoelectric point (pI), and grand average of hydropathicity (GRAVY) of ClaPEPCK, were predicted by the ProtParam tool (https://web.expasy.org/protparam/).^27^

### Bioinformatics Analysis of the ClaPEPCK Gene Family

The gene duplication and syntenic relationship between the *ClaPEPCK* gene family and *Arabidopsis* were established using TBTools with E-value of <e^−10^. The syntenic gene pairs were visualized using the circos tool. The nonsynonymous (Ka) and synonymous substitution (Ks) rates and their ratio (Ka/Ks ratio) for duplicated gene pairs were calculated in the TBtools.^25^ The multiple expectation maximization for motif elicitation (MEME) (https://meme-suite.org/meme/tools/meme.) tool was employed to analyze the de novo motifs in *ClaPEPCKs*.^28^ Multiple sequence alignments were carried out using *ClaPEPCK* along with *AtPEPCK* and Clustal Omega (https://www.ebi.ac.uk/jdispatcher/msa/clustalo).

A phylogenetic tree was then constructed using the aligned sequences obtained from the molecular evolutionary genetics analysis (MEGA v11) program with 1000 bootstraps,^29^ and interactive tree of life (iTOL) v5 (https://itol.embl.de/) was then employed for refinement or visualization.^30^ The gene structure and exon – intron arrangements of the *ClaPEPCK* gene family were analyzed using the Gene Structure Display Server program. About 2 kb upstream sequence of the *ClaPEPCK* was taken for the cis-regulatory element analysis using the PlantCARE tool (https://bioinformatics.psb.ugent.be/webtools/plantcare/html/).^31^

### Subcellular Localization

The CELLO v.2.5 was used to predict ClaPEPCK subcellular localizations (http://cello.life.nctu.edu.tw/).^32^ The construction method of GFP vector is slightly modified according to reference.^33^ The fusion expression vector 35S:*ClaPEPCK3*-GFP and 35S:*ClaPEPCK4*-GFP were constructed using the one-step cloning method (Table S2), and the vector was introduced into *A. tumefaciens*. The inner epidermis of the onion was infected with *A. tumefaciens* for 20 min, cultured on an MS medium overnight for 24 h, washed with sterile water, and prepared for observation under a fluorescence microscope (LUMPUS IX73, China, Shanghai).

### Watermelon Genetic Transformation

As previously described, the genetic transformation was conducted with a few minor modifications. Constructed two overexpression vectors 35S:*ClaPEPCK3*-OE and 35S:*ClaPEPCK4*-OE (Table S2). And constructed four CRISPR vectors 35S:*ClaPEPCK3*#2, 35S:*ClaPEPCK3*#2, 35S:*ClaPEPCK4*#1, and 35S:*ClaPEPCK4*#2 (Table S3). Watermelon cultivars “V603” (*Citrullus lanatus* subsp. *vulgaris*) were used in transgenic experiments, which were self-pollinated for 10 generations to obtain stable phenotypes since 2014.^34^ Watermelon seeds were sown on a 0.6% agar solid medium for 2 d in the dark after being surface-sterilized.^35^ Cotyledons containing growth points were precisely excised to serve as explants that were subjected to infection by *A. tumefaciens* strains harboring the indicated binary vectors. They were then cocultivated with the cotyledon fragments in the dark for 3 d on an MS solid medium containing 1 mg/L 6-Benzylaminopurine (6-BA). Subsequently, the cotyledon fragments were transferred onto a selective induction medium containing 1 mg/L 6-BA, 100 mg/L Kanamycin (Kan), and 250 mg/L Cephalosporin (Cep). The regenerated adventitious buds were excised and transferred onto an elongation medium containing 0.2 mg/L Kinetin (KT), 250 mg/L Cep, and 50 mg/L Kan. The elongation plants were transferred to a rooting medium containing 2 mg/L IBA and 100 mg/L Cep.^36^ Positive transgenic plants were detected using PCR.

## Results

### Bioinformatics Analysis of ClaPEPCK Gene Family

**Figure 1.**
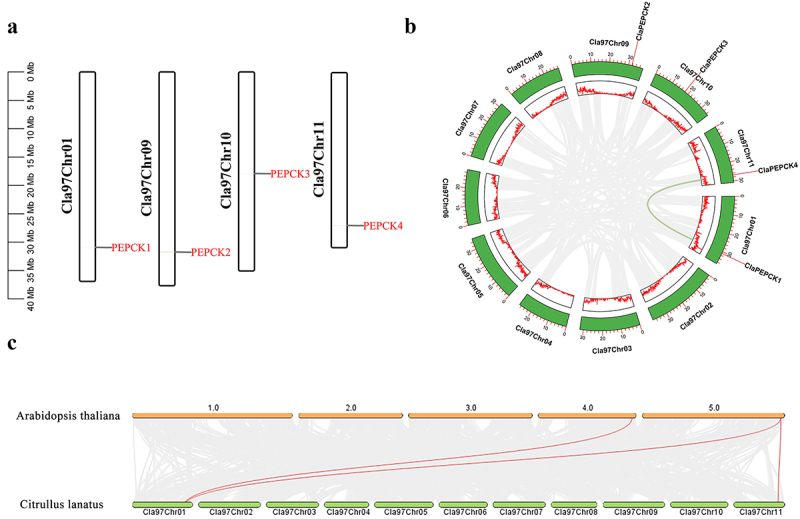
The position of the *ClaPEPCK* gene on chromosomes and its collinearity analysis in watermelons. (a) *ClaPEPCK1*- *ClaPEPCK4* gene distributed on chromosomes. (b) Collinearity analysis between the *ClaPEPCK* gene family. (c) Collinearity analysis of *Arabidopsis PEPCK* genes and watermelon *ClaPEPCK* gene family.

To identify the *ClaPEPCK* gene family, the *PEPCK* pafm domain was acquired from InterPro, after which we identified four *PEPCK* gene family members from the watermelon genome database, named *ClaPEPCK1* to *ClaPEPCK4* (*Cla97C10G192070.1*, *Cla97C10G178710.1*, *Cla97C11G220850.1*, *Cla97C01G017350.3*) with their distribution on the chromosomes (Fig. 1a). Protparam was used to predict the peptide properties of ClaPEPCK. Predicted isoelectric points (pI) for ClaPEPCK proteins were 6.21–9.11, with the molecular weight of *ClaPEPCK* varying between 62.16 and 74.64 KDa. A negative GRAVY score was found for all identified *ClaPEPCKs*, suggesting that they were hydrophilic. Subcellular localization predictions showed that most ClaPEPCK proteins localized in the cytoplasmic, except for ClaPEPCK2 in the plasma membrane (Table S4).

To reveal the expansion of the *ClaPEPCK* gene family, gene duplication events and synteny analysis were also performed (Fig. 1b). Possible duplicate gene pairs *ClaPEPCK1* and *ClaPEPCK4* were used to calculate the ka/ks ratio, which denotes the evolutionary distance between pairs of genes. The ka/ks ratio was NaN, which showed that the evolutionary distance between the two genes was very far. Furthermore, *ClaPEPCK*–*AtPEPCK* collinearity analysis performed to obtain better insights into the evolution of *PEPCK* genes in watermelon revealed that two *ClaPEPCKs* shared collinearity with *AtPEPCK*, suggesting that they could be orthologous (Fig. 1c). The cis-regulatory element analysis of the *ClaPEPCK* gene family also revealed several classes of cis-elements, including light, plant hormones, and stress response elements (Fig. S1). GO analysis revealed that the *ClaPEPCK* gene family primarily contributes to molecular functions and biological processes. Three GO terms had gluconeogenesis (GO:0006094), phosphoenolpyruvate carboxykinase (ATP) activity (GO:0004612), and ATP binding (GO:0005524).

**Figure 2.**
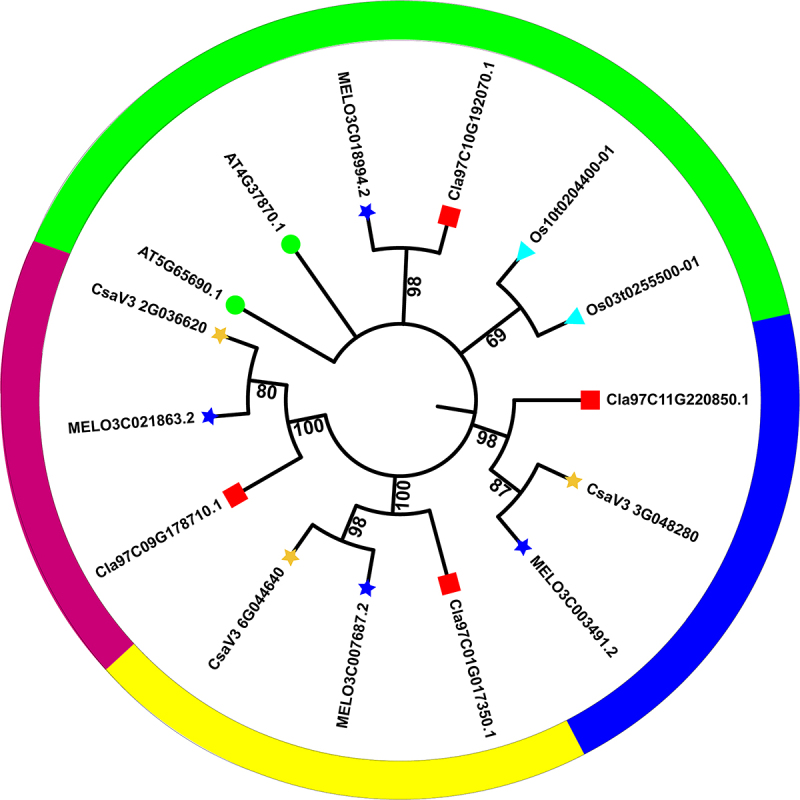
Phylogenetic analysis of ClaPEPCK1–ClaPEPCK4 and its homologues. Cla, watermelon; AT, *Arabidopsis*; Os, rice; MELO, melon; Cs, cucumber. ClaPEPCK1–ClaPEPCK4 are highlighted with red dots. Full-length protein sequences were aligned using ClustalW, and a neighbor-joining phylogenetic tree was constructed using MEGA6 software. Numbers on branches indicate bootstrap percentages for 1000 replicates.

The *ClaPEPCK* gene family conservative motifs were analyzed on the MEME website. *ClaPEPCK2* contained only conserved motif 6, while all other family members contained 10 conserved motifs (Fig. S2). The phylogenetic trees were made with the *PEPCK* of *Arabidopsis*, melon, watermelon, cucumber, rice, corn, tomato, melon, and Physcomitrella patens by using neighbor joining method^29^ (Fig. 2). The phylogenetic tree showed that the *PEPCKs* in the selected species can be divided into seven groups. Among them, the *ClaPEPCKs* are distributed in the first, third, and fourth groups. Phylogenetic analysis showed that *ClaPEPCK1* (*Cla97C01G017350.3*), which are most similar to *MELO3C007687.2* (91.28% sequence identity), cluster together with *Csa V32G036620* and *MELO3C007687.2*. *ClaPEPCK2* (*Cla97C09G178710.1*), which are most similar to *MELO3C021863.2* (88.95% sequence identity), cluster together with *MELO3C021863.2* and *CsaV32G036620*. *ClaPEPCK3* (*Cla97C10G192070.1*), which are most similar to *MELO3C018994.2* (92.00% sequence identity), cluster together with *MELO3C018994.2*. *ClaPEPCK4* (*Cla97C11G220850.1*), which are most similar to *MELO3C003491.2* (93.00% sequence identity), cluster together with *MELO3C003491.2* and *CsaV33G048280*.

### Clone and Obtain Three ClaPEPCK Genes

In order to further investigate the function of *PEPCK* genes, *ClaPEPCK* genes were cloned, namely *ClaPEPCK1* (1962 bp), *ClaPEPCK3* (1983 bp), and *ClaPECPK4* (2019 bp). The corresponding agarose gel electrophoresis results are shown in Fig. S3. But the *ClaPECPK2* gene has not been cloned. This is likely due to the genomic diversity of different watermelon germplasm resources. Therefore, a total of three genes were cloned using this experimental material in this study.

### Nutritional Characteristics of Watermelon Flesh at Different Developmental Stages

**Figure 3.**
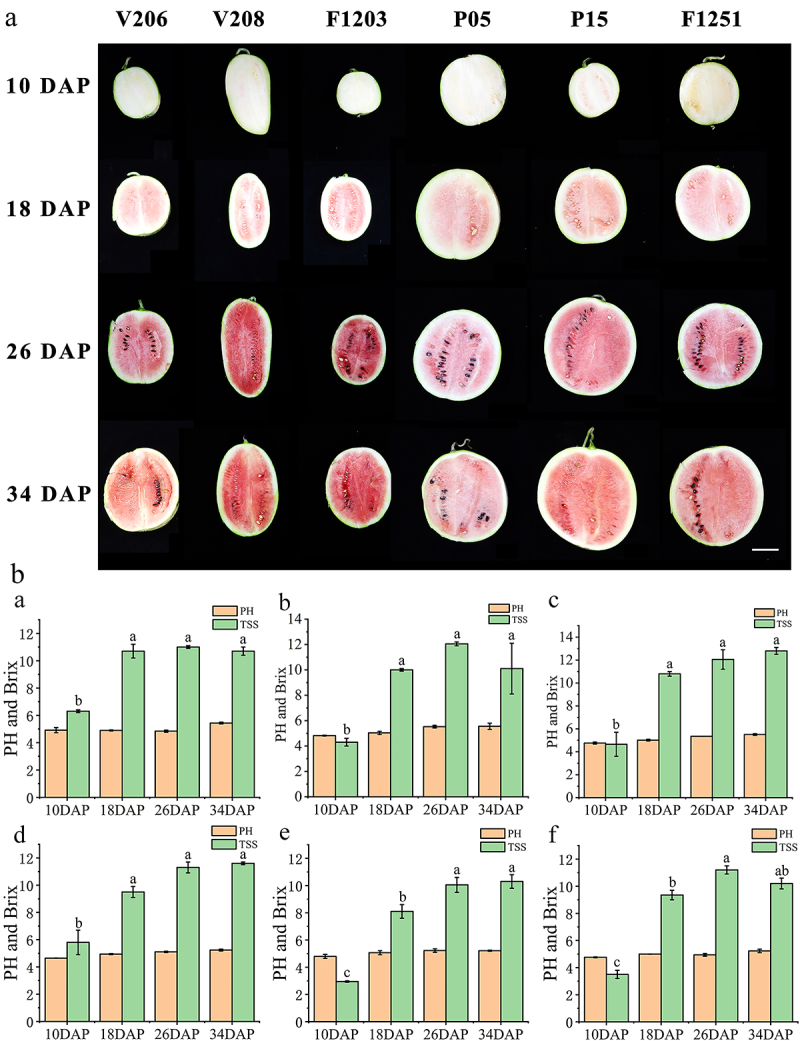
Development stage and physiological indicators of watermelon after pollination. (A) Watermelon fruit of V206, V208, F1203, P05, P15, and F1251 at four developmental stages (bar = 5 cm). (B) Changes of pH and total soluble solids in watermelon of V206 (a), V208 (b), F1203 (c), P05 (d), P15 (e), and F1251 (f) development stage. Error bars show mean ± standard error significance is based on Student’s t-test analysis, *p < .05.

Total soluble solids (TSS) and pH serve as critical indicators of watermelon flesh quality. We measured the TSS and pH of six watermelon varieties at four growth nodes (Fig. 3a). The TSS content was very low at 10 DAP and accumulated rapidly at 18, 26, and 34 DAP (Fig. 3b). The TSS increased from 6.3, 4.3, 4.65, 5.8, 2.9, and 3.5 to 10.7, 12.1, 12.8, 11.6, 10.3, and 10.2 in V206 (Fig. 3a,b), V208 (Fig. 3 B b), F1203 (Fig. 3 B c), P05 (Fig. 3 B d), P15 (Fig. 3 B e), and F1251 (Fig. 3 B f), respectively. The pH changes were only slight during the growth stage. In V206 (Fig. 3a,b), V208 (Fig. 3a,b), and F1203 (Fig. 3 B c), the pH values were 4.92, 4.82, and 4.76 at 10 DAP and 5.43, 5.55, and 5.50 at 34 DAP. In P05 (Fig. 3b.d), P15 (Fig. 3b,e), and F1251 (Fig. 3b,f), the values were 4.65, 4.79, and 4.76 at 10 DAP and 5.24, 5.21, and 5.23 at 34 DAP, respectively.

### The Malate Content and Expression Level of ClaPEPCK in Watermelons

The malate and citric acid contents and the relative expression levels of *ClaPEPCK1*, *ClaPEPCK3*, and *ClaPEPCK4* in watermelon fruits of V206, V208, and F1203 varieties were detected at 10, 18, 26, and 34 DAP. V206 is a germplasm of small fruit watermelon, V208 is a germplasm of large fruit watermelon, and F1203 is a hybrid of V206 and V208 F_1_ generation.

Malate content gradually accumulated in V206 at 26 DAP and then began to decline, while in V208 and F1231, it peaked at 18 d and then gradually decreased. Citric acid content in watermelon fruits of V206, V208, and F1203 increased at 10–18 d, decreased at 18 to 26 DAP, and increased at 26 to 34 DAP. The malate content in fruit gradually accumulated in P05, P15, and F1251, peaking at 18 d and then gradually decreasing. The malate and citric acid contents and the relative expression levels of *ClaPEPCK1*, *ClaPEPCK3*, and *ClaPEPCK4* in watermelon fruits of the P05, P15, and F1251 varieties were detected at 10, 18, 26, and 34 DAP. The citric acid content of P05 increased at 10–18 d, decreased at 18–26 d, and increased at 26–34 d, with this content in P15 and F1251 gradually increasing at 10–34 d. To sum up, the variation trend of the malic acid content in F1 watermelon is basically consistent with that in the male parent.

**Figure 4.**
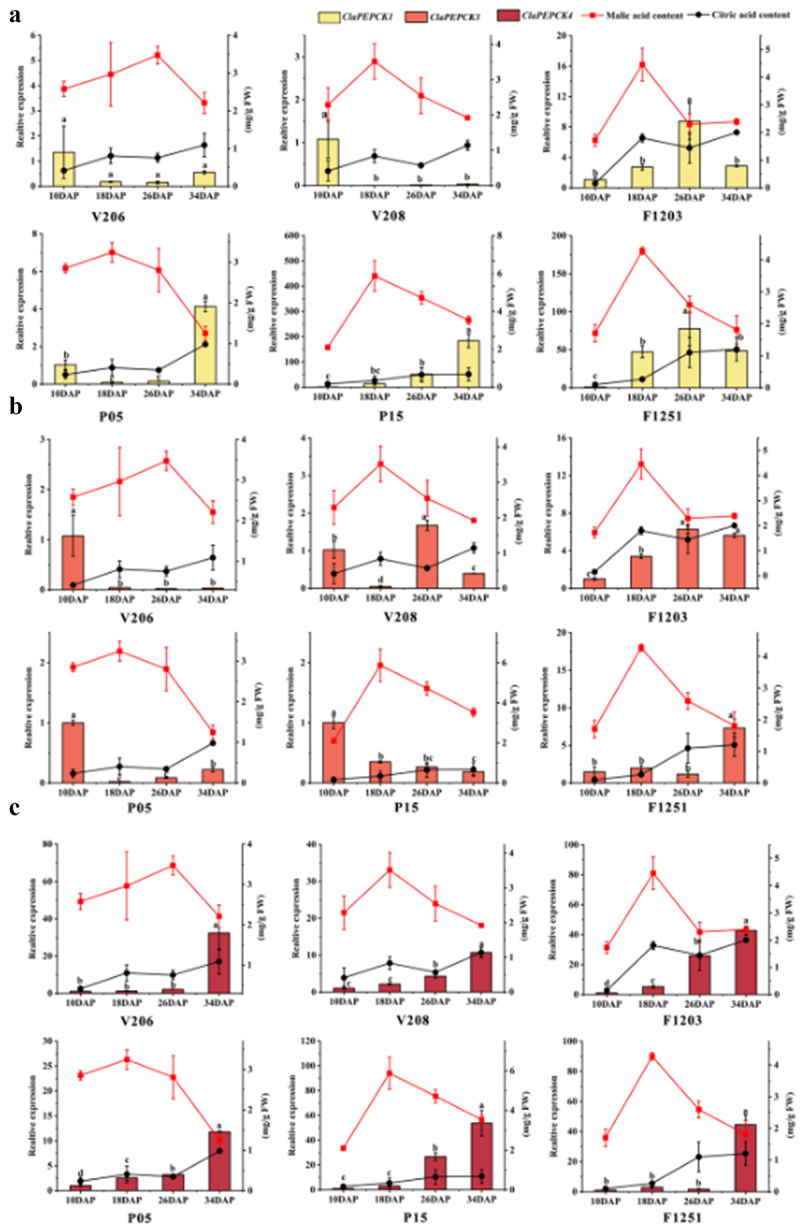
Changes in malic acid and citric acid content and *ClaPEPCK1*, *ClaPEPCK3*, and *ClaPEPCK4* during watermelon development. (a) The relative change in the expression of *ClaPEPCK1* during V206, V208, F1203, P05, P15, and F1251 development. (b) The relative change in the expression of *ClaPEPCK3* during V206, V208, F1203, P05, P15, and F1251 development. (c) The relative change in the expression of *ClaPEPCK4* during V206, V208, F1203, P05, P15, and F1251 development. Error bars show mean ± standard error significance is based on Student’s t-test analysis, *p < .05.

The *ClaPEPCK1* levels in watermelon fruits in V206 and V208 peaked at 10 DAP, while the level in F1203 peaked at 26 DAP. The *ClaPEPCK1* levels in P05 and P15 peaked at 34 DAP, with this level peaking at 26 DAP in F1251 (Fig. 4 A). While the *ClaPEPCK3* level in V206 peaked at 10 DAP, it also peaked at both 10 and 26 DAP in V208, and F1203 exhibited relatively high levels at 26 and 34 DAP. The *ClaPEPCK3* levels in P05 and P15 peaked at 10 DAP, with that of F1251 peaking at 34 DAP (Fig. 4 B). The *ClaPEPCK4* levels in V206, V208, and F1203 gradually increased from 10 to 34 DAP. The *ClaPEPCK4* levels in P05, P15, and F1251 watermelon fruits peaked at 34 DAP (Fig. 4 C). In summary, the expression level of *ClaPEPCK1* gene is very low, and there is no significant difference in V206. Its expression is negatively correlated with the trend of malic acid changes during fruit development in P05. The *ClaPEPCK3* gene is mainly expressed at 10 DAP, 26 DAP, and 34 DAP. The expression level of *ClaPEPCK3* gene is also very low. The expression of ClaPEPCK3 during fruit development is negatively correlated with the trend of malic acid in P15. ClaPEPCK4 showed relatively high expression of 26–34 DAP during fruit development and was negatively correlated with the trend of malic acid, indicating that the *ClaPEPCK4* gene regulates the metabolism of malic acid in the later stage of fruit development. Therefore, selecting the *ClaPEPCK3* and *ClaPEPCK4* for in-depth research. To sum up, the expression trend of *ClaPEPCK4* gene in F1 watermelon is basically consistent with that of the male parent and is also consistent with the changing trend of its malic acid content.

### Subcellular Localization of ClaPepcks

**Figure 5.**
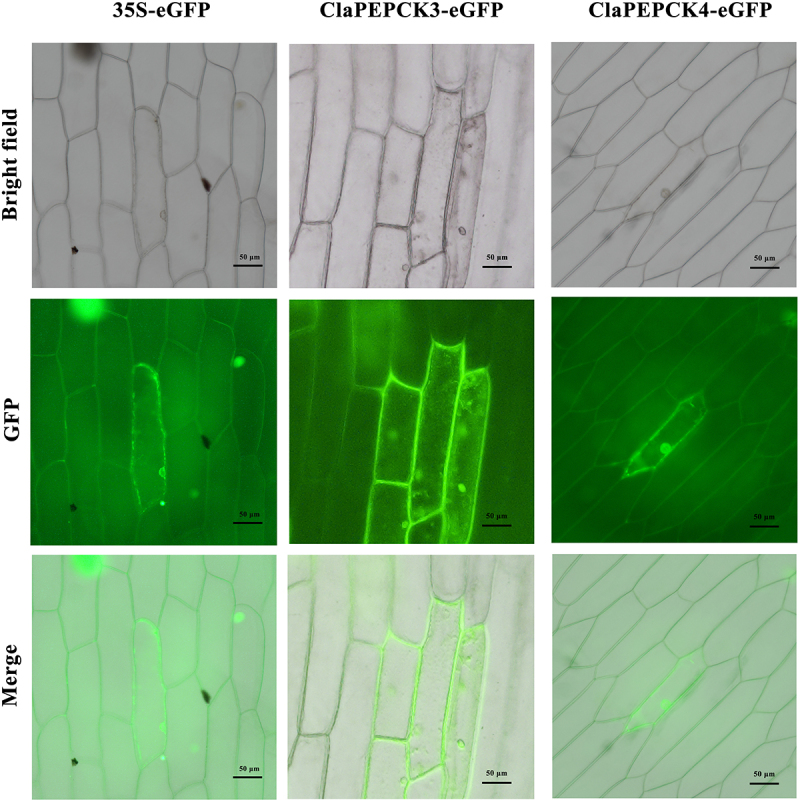
Subcellular localization of the ClaPEPCK3 and ClaPEPCK4 protein. The first row of pictures shows light microscope micrographs of 35S:eGFP, 35S:ClaPEPCK3-gfp, and 35S:ClaPEPCK4-gfp. The second row of pictures shows GFP fluorescence microscope micrographs of 35S:eGFP, 35S:ClaPEPCK3-gfp, and 35S:ClaPEPCK4-gfp. The third row of pictures shows the merged images. The bars represent 50 μm.

To explore the specific role of the ClaPEPCK3 and ClaPEPCK4 protein in the cell, we constructed a vector that fused green fluorescent protein with the ClaPEPCK3 and ClaPEPCK4 protein. The constructed vector was transferred into *A. tumefaciens* to infect the inner epidermal cells of the onion for 24 h, which was then prepared and observed under a fluorescence microscope. Our observation revealed fluorescence originating from the GFP protein fused with ClaPEPCK3 and ClaPEPCK4 in both the cytoplasm and nucleus. These indicate that ClaPEPCK3 and ClaPEPCK4 exert its function within these cellular compartments. As predicted, PEPCK catalyzes oxaloacetic acid decarboxylation to phosphoenolpyruvate in the cytoplasm (Fig. 5).

### ClaPepck4 Significantly Affects Regulates Malic Acid Metabolism in Watermelon

**Figure 6.**
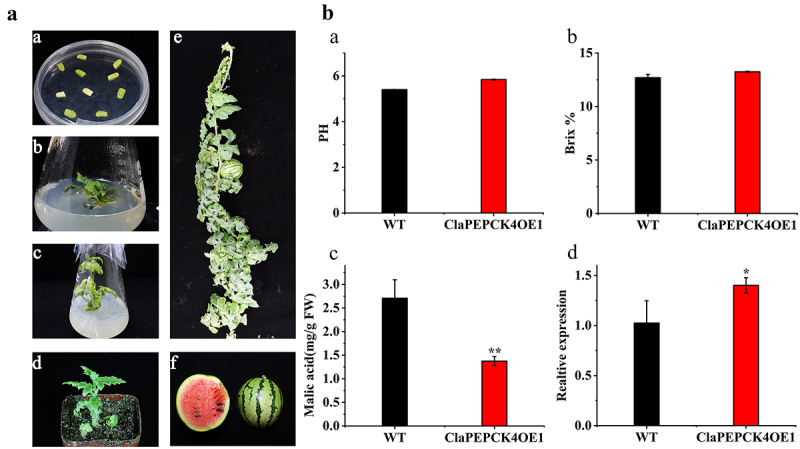
*ClaPEPCK4* overexpression triggers decreased malic acid content in watermelon. (A) Transformation of watermelons overexpressing the *ClaPEPCK4* gene. (B) The average value of pH (a), TSS (b), malic acid content (c), and relative gene expression levels (d) of overexpressing *ClaPEPCK4* watermelon fruits at 34 DAP. Error bars show mean ± standard error significance is based on Student’s t-test analysis, *p < .05.

To explore the role of the *ClaPEPCK* gene in watermelon, we constructed a vector with overexpression of the *ClaPEPCK* gene and infected watermelon cotyledon with *A*. *tumefaciens* to obtain six T_0_ *ClaPEPCK3* overexpressing transgenic plants and four T_0_ *ClaPEPCK4* overexpressing transgenic plants. One of T_0_ ClaPEPCK4 overexpressed plants (Fig. 6 A a, b, c, d) and one of T_1_ ClaPEPCK4 overexpressed plants (Fig. 6 A e, f) were shown, which were the most representative. *ClaPEPCK4* overexpression in the transgenic watermelon fruits leads to decreased malic acid content and increased soluble solid content at 34 DAP (Fig. 6 B a, b, c). The expression levels of *ClaPEPCK4* in fruits of overexpressed transgenic plants were measured, revealing a notable increase in *ClaPEPCK4* expression (Fig. 6 B d). This means that the *ClaPEPCK4* gene contributes to gluconeogenesis in watermelon fruits. The expression levels of *ClaPEPCK3* in fruits of overexpressed transgenic plants were measured, revealing a notable increase in *ClaPEPCK3* expression. However, *ClaPEPCK3* is not as important as *ClaPEPCK4* which leads to a decreased malic acid content significantly (Fig S4 A, B).

**Figure 7.**
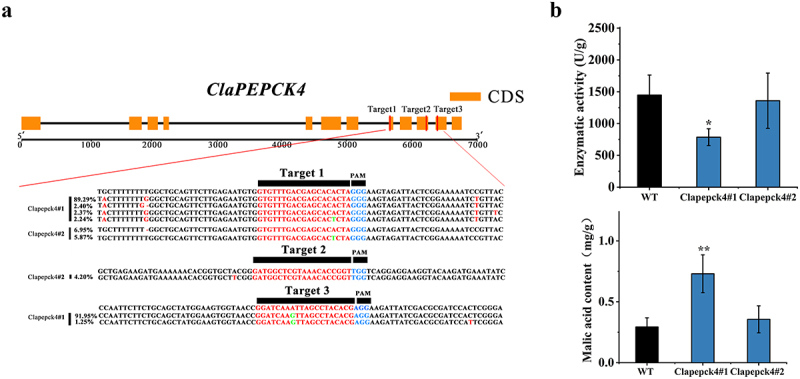
Gene editing *ClaPEPCK4* affects malic acid content and stomatal movement. (a) Editing *ClaPEPCK4* gene efficiency and mutation types. (b) The average value of the malic acid content and enzyme activity in leaves of genetically edited plants which have grown for 60 d. Error bars show mean ± standard error significance is based on Student’s t-test analysis, *p < .05, ***p < .001.

We performed gene editing on the *ClaPEPCK4* gene and found multiple SNP mutations in the *ClaPEPCK4* gene in the gene editing-positive plants, which were the two most efficient knockout T_0_ plants (Fig. 7 A). The average value of the malate content in the leaves of 60-d-old transgenic plants was markedly higher than that observed in the leaves of 60-d-old transgenic plants (Fig. 7 B). The *ClaPEPCK4* gene in the gene editing-positive watermelon plants is less likely to bear fruits, resulting in an inability to obtain self pollinated watermelons. Two Clapepck4#1 strains and three Clapepck4#2 strains were ultimately obtained through genetic transformation (Fig S5 A). Interestingly, gene-edited plants displayed larger stomatal openings in darkness than the wild type did. Additionally, both transgenic and non-transgenic plants showed difficulty closing their stomata after 4 h darkness (Fig S5 A, B).

## Discussion

The pH, soluble solids, malate, and citric acid levels in the fruit are pivotal factors influencing the flavor profile of watermelon. However, research on organic acids in watermelon fruits has primarily focused on identifying the types and measuring the contents of these acids in mature fruits. Therefore, studying the molecular mechanisms of malic acid metabolism throughout watermelon development holds paramount importance for enhancing its flavor through molecular breeding.

The flesh of watermelon at different developmental stages has the following nutritional characteristics. Firstly, investigations of the variations in both edge and central soluble solids across six varieties showed that the rapid accumulation of soluble solids in fruits occurred between 10 and 26 DAP during fruit development. Subsequently, the accumulation of soluble solids slowed down 26–34 DAP. It was reported that fruit development occurred mainly 2 weeks after flowering, and the length and weight of the fruits changed rapidly in cucumbers.^37^ Studies using apples uncovered that fructose concentration increased rapidly at 4–12 weeks after flowering until it remained unchanged during fruit harvest; sucrose did not accumulate rapidly until 6–8 weeks after flowering but increased until fruit harvest; glucose and galactose concentrations peaked at 6 weeks after flowering, decreased at 6–10 weeks after flowering, and then remained almost unchanged during fruit harvest; starch concentration peaked within 6–10 weeks after flowering and then decreased at fruit harvest.^8^ To sum up, the main sugars found in melons are sucrose, glucose, and fructose, with sucrose content in the pulp peaking sharply from 23 to 35 DAP and decreasing at fruit ripening, while glucose and fructose levels change slightly during development. Secondly, the malate content in all six varieties surpassed that of citric acid across each period, providing further evidence that watermelon is a malolactic fruit. The malate and citric acid contents differed in the selected watermelon varieties, but the trends were almost the same, accumulating malate and citric acid in the early stage of fruit development and decreasing in the late stage, which was consistent with the organic acid accumulation pattern in crown pear, pineapple, and dragon fruit^38–40^ In the apple cultivar “Honey Crisp,” the concentrations of malate, succinic acid, fumaric acid, and maleic acid increased within 2 to 4 weeks after flowering and then gradually decreased to fruit harvest, where the concentrations of citric, quinic, ascorbic, and shikimic acids decreased from 2 weeks after flowering until fruit harvest.^8^ Different fruits derive unique flavors from various specific organic acids. The main organic acid in mangoes, strawberries, pineapples, and citrus is citric acid, while that in apples, loquats, peaches, and bananas is malate. Malate and citric acid are important organic acids in watermelon flesh, which make sarcocarp a special flavor.^41^ The variation in organic acid content is a complex quantitative trait governed by multiple genes.^42^ During watermelon evolution, malate content in watermelon fruits decreased gradually from the wild type to the cultivated type, while citric acid content was the opposite.^43^ To sum up, during the watermelon growth period, malate content first increased and then decreased. Thirdly, the variation trend of the malic acid content in F_1_ watermelon is basically consistent with that in the male parent. Previous studies have mainly found that the sugar in watermelon of hybrid offspring is greatly influenced by the maternal parent.^44,45^ It was reported that the contents of acid in the fruit pulp of citrus triploid hybrids were more influenced by the male parent.^46^ However, the contents of acid in the fruits of mango hybrid progenies were prone to be affected by the female parent, the inheritance of the carotenoids was inclined to the high-value parent and was greatly influenced by the male parent.^47^ In fact, exploring the genetic characteristics of acid content in hybrid watermelon offspring is also crucial for efficient and precise breeding.

PEPCK serves as a crucial enzyme in plant growth and development. Phylogenetic analysis of ClaPEPCK revealed a relatively conserved *PEPCK* gene in Cucurbitaceae. Conserved domain analysis showed that most ClaPEPCK retained intact domains, indicating their importance in watermelon and potential significant functions. To investigate the impact of these genes on malate and citric acid during watermelon fruit development, we analyzed the relative expression levels of genes associated with malate and citric acid across six watermelon varieties. We observed that the malate content initially increased before decreasing, whereas the citric acid content exhibited a gradual rise. Notably, the malate content consistently exceeded that of citric acid. A study of five organic acids in the ripe fruit of the “Charles ton Gray” and “Jubilee” varieties was consistent with our findings that malate was predominantly organic in all parts.^41^ *ClaPEPCK1* and *ClaPEPCK3* levels declined during malate accumulation and markedly increased as malate decreased. Similarly, ClaPEPCK4 exhibited a corresponding trend across the selected varieties: decreasing as malate accumulated in the early fruit development stages and significantly increasing as malate content declined in later stages. These results indicate that *ClaPEPCK1*, *ClaPEPCK3*, and *ClaPEPCK4* expression are inversely proportional to malate content variations in fruits. The *ClaPEPCK* gene is involved in malate metabolism in watermelon fruit, and *ClaPEPCK4* may be the key gene of malate metabolism in fruit. In the detection of gluconeogenesis-related enzymes in fruits, PEPCK was found to be present in blackberry, blueberry, cherry, grape, plum, and tomato fruits, and the abundance of PEPCK increased significantly during the ripening process of tomato fruits, indicating that PEPCK played a role in gluconeogenesis in most fruits.^18^ Cis-elements play a central role in regulating biological development, and changes in acclimation traits are caused by the diversity of cis-acting elements in domestication genes during crop domestication.^48^ The analysis of the cis-acting elements of *ClaPEPCK* showed that all genes had numerous photoresponsive elements, indicating their expression was regulated by light. C4 plants can be classified into NADP-ME, NAD-ME, and PEPCK types according to their major decarboxylase enzymes of tetracarbonate in vascular sheath cells. As a C4 plant, watermelon has not been studied to prove its main decarboxylase. NADP-ME serves as the primary decarboxylase enzyme in maize, while PEPCK acts as a co-decarboxylase in various other species. This indicates that the decarboxylation of tetracarbonate within the vascular sheath cells is a multifaceted process involving more than just a single enzyme.^49^ As the cis-acting elements of *ClaPEPCK* all have elements that respond to abscisic acid, abscisic acid may inhibit seed germination by regulating *ClaPEPCK* expression.

*ClaPEPCK4* in watermelon may be a key gene that promotes malate metabolism and gluconeogenesis in fruits. The *ClaPEPCKs* gene was expressed in both the nucleus and cytoplasm, indicating its role in these compartments. The expression levels of ClaPEPCK4 in fruits of overexpressed transgenic plants reveal a notable increase, which leads to a significantly decreased malic acid content. This was consistent with findings from previous studies. Overexpression of SlPEPCK in tomato fruits enhanced the growth of seedlings, increased soluble sugar, and decreased malate content in ripe fruits; *PEPCK* markedly contributed to the regulation of the malate content in fruit ripening.^14^ In the evaluation of transgenic plants, interesting findings emerged. Stomata, crucial for plant transpiration and photosynthesis, comprise two guard cells and markedly contribute to disease resistance and response to drought and other stresses. *ClaPEPCK4* knockout transgenic plants exhibited larger stomatal openings 1 h under dark conditions, unlike normal plants, which showed smaller stomata. This suggests that *ClaPEPCK4* is involved in regulating stomatal closure in watermelon leaves. It was reported that malate metabolism affected stomatal opening in tomatoes.^50^ Aluminum-mediated malate transporters can control stomatal opening and closure through malate in tomato and *Arabidopsis* guard cells (Sasaki et al., 2022). *AtABCB14* regulates stomatal motility by transporting malate from apoplast to guard cells, thereby increasing osmotic pressure.^51^ It is likely that the decrease in expression level of *ClaPEPCK4* leads to changes in malate-mediated stomatal movement, adjusting the opening and closing of stomata to regulate gas exchange and water loss.

As living standards improve, the development of healthy new crop varieties becomes a key focus in breeding. This study explores the molecular regulatory mechanisms of organic acids in watermelons, offering genetic targets for breeding new varieties that are sweeter yet contain lower sugar content.
